# Ionic Conductive Membranes for Fuel Cells

**DOI:** 10.3390/membranes11030159

**Published:** 2021-02-25

**Authors:** Riccardo Narducci

**Affiliations:** Department Industrial Engineering and International Laboratory: Ionomer Materials for Energy, University of Rome Tor Vergata, 00133 Roma, Italy; riccardo.narducci@uniroma2.it

The need to reduce pollution and the continuous increase in petrol cost have reinforced the interest in fuel cells (FCs), efficient and clean systems for the conversion of fuel into energy. Polymer electrolyte membrane fuel cells (PEMFCs) exhibit excellent characteristics in their weight, volume, and current density for automotive applications and cogeneration systems. Unfortunately, the high cost of perfluorinated membranes and the low stability of anionic membranes in an alkaline environment still limit their use. During the past years, this kind of membranes has been widely explored in terms of synthesis of new materials, grafting of strong and stable functional groups, mechanical properties, conductivity, FC performance, and so forth. This special issue of Membranes is dedicated to this exciting research field, with some excursions in related fields, focusing on commercial polymers, like Nafion^®^ and Aquivion^®^, and promising and low-cost sulfonated aromatic polymers (SAPs) and poly(vinyl alcohol) (PVA) and some strategies to enhance stability, like cross-linking (XL), increase crystallinity with the use of high boiling solvents or nanocomposites. All of these properties were studied with classical investigation techniques, like impedance or the new Ionomer n_c_ Analysis (INCA) method.

To reflect the broad scope of this topic, contributions from leading scientists across the world, whose research addresses ionomeric membranes from different perspectives, sharing a common vision of pollution reduction and the search for sustainable energy sources, have been gathered. Giancola et al. [1] investigated the possibility to increase the working temperature and endurance of short-side-chain perfluorosulfonic acid (SSC-PFSA) Aquivion^®^ membranes with an annealing procedure in the presence of the plasticizing solvent DMSO. It was applied for the first time through the Ionomer n_c_ Analysis (INCA) method in order to evaluate ionomer thermomechanical properties and to probe the increase of crystallinity during the annealing treatment. Complementary differential scanning calorimetry (DSC) and dynamic mechanical analysis (DMA) measurements confirmed the increase of polymer stiffness over the whole range of temperature. 

Akrout et al. [2] focused on the use of bifunctional nanoclay halloysites, grafted with amino groups and embedding with radical scavengers, that is, CeO_2_ nanoparticles, to form composite membranes with Aquivion^®^ ionomer. The composite with 4 wt% of CeO_2_@HNT-NH_2_ showed unchanged tensile properties but presented high proton conductivity and increased stability to radical attack compared with nonmodified Aquivion^®^. 

Avci et al. [3] studied salinity gradient power (SGP) harvesting by reverse electrodialysis (RED) with Nafion 117 and 115 membranes for NaCl and NaCl + MgCl_2_ solutions in order to measure the gross power density extracted under high salinity gradient and to evaluate the effect of Mg^2+^ (the most abundant divalent cation in natural feeds) on the efficiency in energy conversion. In all tests, Nafion 117 exhibited superior performance when 0.5/4.0 M NaCl was fed through 500 µm thick compartments at a linear velocity of 1.5 cm·s^−1^. However, the gross power density of 1.38 W·m^−2^ detected in the case of pure NaCl solutions decreased to 1.08 W·m^−2^ in the presence of magnesium chloride. Although Nafion membranes exhibited better performance than CMX and Fuji-CEM-80050, their use is limited by high cost, and a significant reduction of membrane price is required for affordable RED applications. 

Kim et al. [4] focused on cross-linked sulfonated polyphenylsulfone (CSPPSU) with high ion exchange capacity (IEC) and developed an activation process treatment with alkaline and acidic solutions to remove sulfur dioxide (SO_2_), which forms as a byproduct during heat treatment. The membranes obtained using this activation method had high thermal, mechanical, and chemical stabilities. In I-V_iR free_ studies for fuel cell evaluation, high performances similar to those using Nafion were obtained, and by using a constant current method, a stability of 4000 h was attained. 

Schiavone et al. [5] described the microstructural characterization by small-angle neutron scattering (SANS) method of sulfonated sPS films and sPS–fullerene composite membranes at different temperatures between 20 and 80 °C under a relative humidity (RH) level of 10% to 70%. The water is taken up around the agglomerations of sulfonic groups and gives rise to hydrated domains and grows in size and number by increasing the hydration level, by increasing the temperature at a constant hydration level; due to desorption of some water, these domains shrink, mostly from the bulk amorphous regions. The sulfonated sPS–fullerene composite membranes perform at a high temperature much better than the fullerene-free membranes in terms of proton conductivity in liquid water. Apparently, this may be related to the formation of additional hydrated pathways. 

Marf et al. [6] focused on the preparation and characterization of polymer blends based on poly(vinyl alcohol) and chitosan (PVA/CS) incorporated with various quantities of ammonium iodide. Structural analysis from X-ray diffraction (XRD) revealed structural change upon the addition of NH_4_I salt. The protruded appearance on the samples’ surface was evidently shown at high salt concentrations (~50 wt %) in the field-emission scanning electron microscopy (FE-SEM) images. The system incorporated with 40 wt % of NH_4_I salt exhibited a high ion transference number. A potential cutoff of 1.33 V was recorded for the electrolyte system as decomposition voltage. 

A review by Sazali et al. [7] examined recent advances and up-to-date modeling in fuel cell technologies, especially towards polymer electrolyte membrane fuel cells (PEMFCs), solid oxide fuel cells (SOFCs), and direct methanol fuel cells (DMFCs). 

A review by Dickinson and Smith [8] focused on theoretical models and their parameterization used to describe the proton-conductive membrane in polymer electrolyte membrane fuel cells (PEMFCs), especially for Nafion 1100 materials. Detailed attention was given to methods of coupling proton transport with water uptake and diffusive water transport. Other sections addressed the formulation and parameterization of models incorporating interfacial transport resistances, hydraulic transport of water, swelling, and mechanical properties. Lastly, a section was dedicated to the formulation of models predicting the rate of membrane degradation and its influence on PEMFC behavior. 

A review by Umar et al. [9] reported the use of a novel biotechnique called benthic microbial fuel cells (BMFCs), a kind of microbial fuel cells (MFCs) distinguished by the absence of a membrane, for the bioremediation of pollutants and for renewable energy production via different electron pathways. 

I am confident that the articles contained in the Special Issue will serve to further stimulate advances in this research area. I thank all our friends and colleagues who contributed papers to the themed issue.
